# Monovalent Lectin Microvirin Utilizes Hydropathic Recognition of HIV-1 Env for Inhibition of Virus Cell Infection

**DOI:** 10.3390/v17010082

**Published:** 2025-01-09

**Authors:** Bibek Parajuli, Kriti Acharya, Harry Charles Bach, Shiyu Zhang, Cameron F. Abrams, Irwin Chaiken

**Affiliations:** 1 Department of Biochemistry and Molecular Biology, Drexel University College of Medicine, Philadelphia, PA 19102, USA; acharyakriti5@gmail.com (K.A.); harrybach1996@gmail.com (H.C.B.); shiyu.zhang3@gmail.com (S.Z.); 2 Department of Chemical and Biological Engineering, Drexel University, Philadelphia, PA 19104, USA; cfa22@drexel.edu

**Keywords:** SPR, epitope, Biacore, calorimetry, Sensorgram, ELISA, HIV-1, virus, mAbs, glycosylation

## Abstract

Microvirin is a lectin molecule known to have monovalent interaction with glycoprotein gp120. A previously reported high-resolution structural analysis defines the mannobiose-binding cavity of Microvirin. Nonetheless, structure does not directly define the energetics of binding contributions of protein contact residues. To better understand the nature of the MVN-Env glycan interaction, we used mutagenesis to evaluate the residue contributions to the mannobiose binding site of MVN that are important for Env gp120 glycan binding. MVN binding site amino acid residues were individually replaced by alanine, and the resulting purified recombinant MVN variants were examined for gp120 interaction using competition Enzyme-Linked Immunosorbent Assay (ELISA), biosensor surface plasmon resonance, calorimetry, and virus neutralization assays. Our findings highlight the role of both uncharged polar and non-polar residues in forming a hydropathic recognition site for the monovalent glycan engagement of Microvirin, in marked contrast to the charged residues utilized in the two Cyanovirin-N (CVN) glycan-binding sites.

## 1. Introduction

HIV-1 envelope is a protein complex composed of non-covalently associated outer glycoprotein gp120 and transmembrane gp41 subunits [1,2]. Over 22 glycosylation sites have been reported in gp120 and gp41, accounting for nearly half of the mass of the envelope protein [2,3]. These glycosylation patches act as shields, preventing the glycoproteins from proteolytic cleavage and providing them with stability [4]. Interestingly, these glycosylation patches have also been exploited as targets for preventing infection by lectin proteins such as Cyanovirin-N [5], Griffithsin [6], Concanavalin-A [7], Actinohivin [8], and Microvirin [9,10], as well as monoclonal antibodies such as 2G12 [11], PGT antibodies [12], and VRC antibodies [13].

**Figure 1:**
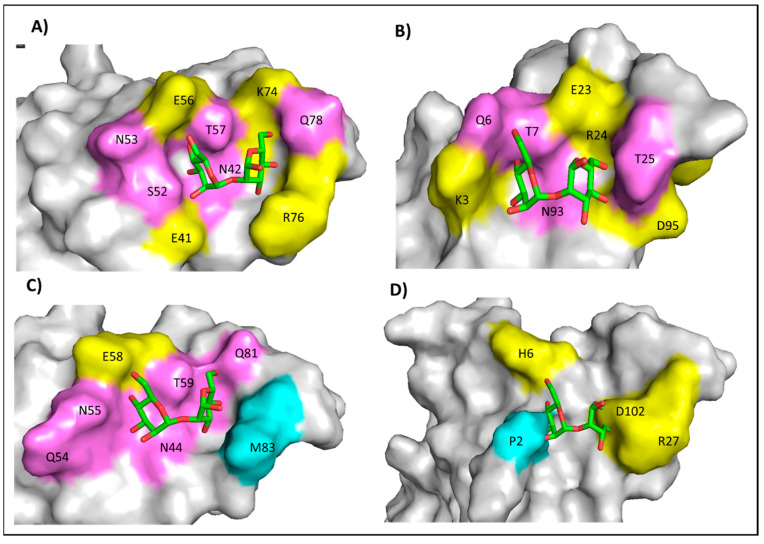
Structural comparison of low-affinity binding site of Cyanovirin-N and Microvirin. (**A**) Figure on the top left represents high-affinity mannobiose-binding site of Cyanovirin-N, shown as surface representation, with (PDB ID: 1IIY) bound to mannobiose shown in green sticks. Four charged residues are highlighted as yellow surfaces (E56, K74, E41, R76). Polar amino acids are shown in magenta (N53, S52, T57, N42, Q78). (**B**) Figure on the top right shows low-affinity mannobiose-binding site of Cyanovirin-N, shown as surface representation, with (PDB ID: 1IIY) bound to mannobiose shown in green sticks. Four charged residues are highlighted as yellow surfaces (K3, E23, R24, D95). Polar amino acids are shown in magenta (N93, T7, Q6, T25). (**C**) Figure in the bottom left shows the high-affinity binding site of Microvirin bound to mannobiose (shown in green sticks, PDB ID: 2YHH). Only one charged amino acid is seen (E58). Polar amino acids are shown in magenta and non-polar amino acids in blue. (**D**) Figure on the bottom right highlights the cavity/domain of Microvirin that corresponds to the low-affinity mannobiose-binding site of Cyanovirin-N (PDB ID: 2YHH; shown in surface representation). Structure shows surface collision of mannobiose (shown in green stick) with charged amino acid Asp102 (yellow) and non-polar amino acid Pro2 residue (blue) from Microvirin low-affinity site. Mannobiose position in the corresponding region was generated by structural overlay of Cyanovirin-N structure (PDB ID: 1IIY) with Microvirin structure (PDB ID: 2YHH), and the mannobiose from CVN structure is highlighted with stick representation. Structures were drawn using Pymol (TM) 3.0.3 (Schrodinger Inc., New York, NY, USA.).

Microvirin is a recently identified lectin that has 31% sequence identity with Cyanovirin-N (Figure S1A) and is structurally very similar to CVN (Figure S1B) [14]. Assessment of the glycan engagement sites of CVN and MVN has also shown a high degree of similarity in residue positioning with subtle differences (Figure S1C). Despite these similarities, MVN has a much simpler gp120 binding pattern (monovalent, 1:1 stoichiometry) [14,15] than Cyanovirin [16]. Structural analysis of Cyanovirin-N shows the presence of two mannobiose binding domains, one of high affinity and the second of low affinity (Figure 1A,B) [17]. Both mannobiose binding sites are rich in charged residues (Figure 1A,B) and have the potential to dually engage two glycosylation sites of glycoprotein gp120 [18]. This behavior enables Cyanovirin-N to have a multivalent interaction with gp120 at a high affinity [18,19]. In addition to CVN, other lectins and antibodies such as Concanavalin-A, Griffithsin, and 2G12 also have significant contents of charged residues (Figure S2 and Table S1) in their active sites. In contrast to CVN, MVN has only one mannobiose-binding domain (Figure 1C). Structural comparison of CVN with MVN shows that the second mannobiose-binding site of MVN is occluded by the presence of proline and aspartate (Figure 1D). Also, the first mannobiose-binding site of MVN has relatively more non-polar and polar residues, with only one charged residue E58 (Figure 1C). Even at its binding interface, >80% of Man-Alpha 1,2 Man-Alpha sits on the polar and non-polar surface, with only one hydrogen-bonding interaction seen with E58 (Figure 1C). We seek in this study to understand how these amino acid differences play a role in MVN engagement and antiviral function.

More importantly, our recently reported protein chimera MVN-DLI, comprising MVN and an MPER-derived peptide, has the ability to irreversibly inactivate HIV-1 virus- [14] and Env-expressing cells [20] by inducing virolysis and membrane destabilization [14,20]. From a mechanistic standpoint, defining the MVN engagement mechanism can help us further understand the mechanism of inactivation of virus- as well as Env-expressing cells. By creating variants of MVN and performing binding analysis, herein, we investigated the amino acids important for the recognition of HIV-1 gp120 by MVN. Our data highlight the importance of polar and non-polar residues in forming a hydropathic surface in MVN for gp120 recognition.

## 2. Materials and Methods

### 2.1. Reagents

Plasmids for MVN were provided by Dr. Carole Bewley’s lab at NIH. Mutagenesis was conducted using the Quick Change II XL Site Directed Mutagenesis kit (Cat. No. 200522), and the reaction was conducted using PFU Ultra Polymerase (Cat. No. 600380-51) provided by Agilent technologies. The DNA was purified using the Promega miniprep kit (Cat. No. A1222). All other reagents for protein purification were purchased from Sigma Aldrich unless otherwise specified. HEK-293F cells were purchased from ATCC. Modified human osteosarcoma cells (HOS.T4.R5) were a gift from Nathaniel Landau. HEK293T cells were purchased from ATCC. Plasmids for transfection and protein production were produced using a Qiagen Maxiprep Kit (Cat. No. 10063). HIV-1 YU2 gp120, HIV-1 YU2 gp160, JRFL gp160, and Bal.01 gp160 plasmids were gifts from Joseph Sodroski.

### 2.2. Generation of MVN Mutants for Binding Studies

Plasmids containing MVN were confirmed for base sequence. The forward primers were as follows:

5′-GCAGTTCGGGGATGCAAACTTCCAAGAAACC-3′

5′-GCAGTTCGGGGATCAAGCCTTCCAAGAAACC-3′

5′-GTGACCATATCGGTGCTATAGATGGGGAATTGCAG-3′

5′-GGATCAAAACTTCCAAGAAGCCTGCCAAGATTGTCG-3′

5′-GGATCAAAACTTCCAAGCAACCTGCCAAGATTGTCG-3′

5′-GGTGTGTACTTGTGCAACAATGGATGGGGAATGG-3′

5′-GGTGTGTACTTGTCAAACAGCGGATGGGGAATGG-3′

5′-GGTGTGTACTTGTCACACACACGATGGGGAATGG-3′

5′-GGTGTGTACTTGTGCAACAGCGGATGGGGAATGG-3′

Their reverse-complement primers were used to construct Q54A, N55A, N44A, T59A, E58A, Q81A, M83A, Q81H/M83H, and Q81A/M83A mutations, respectively, on MVN plasmids using a standard mutagenesis protocol from Agilent technologies. All mutations were validated by sequencing (Genewiz Inc. NJ, USA) post-mutagenesis.

### 2.3. Expression and Purification of MVN, Its Mutants, and the gp120 Protein

Microvirin protein and its mutants were purified based on the protocol reported earlier [14]. The homogeneity of these proteins was assessed with protein eluates run on 18% gel. The gp120 protein was purified using a method described earlier [14].

### 2.4. Surface Plasmon Resonance Interaction Analysis

The kinetic interaction assay was performed using an SPR biosensor, Biacore 3000 (Biacore, Uppsala, Sweden). The SPR experiments were conducted at 25 °C in 1X PBS buffer (1 mM KH_2_PO_4_, 10 mM Na_2_HPO_4_, 137 mM NaCl, and 2.7 mM KCl, pH 7.4) with 0.005% Tween 20. Initially, CM5 sensor chips were activated using a standard amine coupling method using 1-ethyl-3-(3-(dimethylamino)propyl) carbodiimide (EDC) and N-hydroxysuccinimide (NHS) solution. Briefly, carboxyl groups on the sensor surface were activated by injection of 50 µL of a solution of 0.2 M EDC and 0.05 M NHS at a flow rate of 5 µL/min. Next, the protein ligand gp120 was diluted in low-pH solvent (10 mM sodium acetate pH 5.0) and passed over the activated CM5 chip surface for covalent capture for the desired number of response units. Post-gp120 capture, the chip was deactivated using 1 M ethanolamine, pH 8.5. The antibody Vectibix (Amgen; NDC 55513-954-01) was used as a negative control surface since there was no detectable binding to MVN at its highest concentration (50 µM). SPR experiments were performed to measure the binding properties of MVN and its mutants. The real-time interaction was measured by injecting purified recombinant Microvirin and its mutational variants over these surfaces. All the procedures were automated to create repetitive cycles of injection of 0–400 nM of the Microvirin (flow rate 50 µL/min). Surface regeneration was achieved by two 10 μL injections of 15 mM HCl solution at a flow rate of 100 μL/min after the dissociation phase. All analyses were performed in triplicate.

### 2.5. SPR Data Evaluation

The Sensorgram for direct binding was analyzed using BIA Evaluation v4.0 software provided by Biacore (GE Healthcare). Prior to calculation, the binding data were corrected for non-specific interaction by subtracting the reference surface data from the reaction surface data, and were further corrected for buffer effects by subtracting the signals due to buffer injections from those due to protein sample injections. Sensorgram were analyzed by the method described earlier [21]. Individual kinetic parameters were obtained from three independent experiments.

### 2.6. Isothermal Titration Calorimetry (ITC) Binding Analysis of MVN and Its Mutants with gp120 Protein

To confirm our SPR binding data and to understand the thermodynamics of binding, ITC experiments were performed, and the dissociation constant was determined at 25 °C on a VP-Isothermal Titration Calorimeter (VP-ITC) system (Micro Cal TM, GE Healthcare, Freiburg, Germany). In brief, MVN or its mutant protein solutions, dialyzed on 1XPBS, were separately added into (3–5) µM of WT YU2 gp120 dialyzed in 1X PBS. The concentrations of MVN mutants were as follows: MVN WT (30 µM), N44A (400 µM), Q54A (200 µM), T59A (400 µM), Q81H/M83H (300 µM), and Q81A/M83A (400 µM). For null Microvirin variants, ITC analysis was performed at much higher concentrations to rule out any possible binding. Some of these MVN mutants showed limited expression; hence, their ITC analyses were performed at mid-micromolar concentrations. A total of 40 injections were performed, each containing 8 µL MVN. All experiments were performed at 25 °C using 1X PBS buffer at pH 7.4. The resulting heat change upon injection was integrated over a time range of 300 s, and the obtained values were fitted to a standard single-site binding model using Origin.

### 2.7. Production and Characterization of YU2, Bal.01, and JRFL Pseudo-Typed Viruses

Pseudo-viruses were produced by co-transfection of two plasmids: [1] an envelope plasmid that coded for either the YU2, Bal.01, or JRFL gp160 region; and [2] a backbone sequence corresponding to envelope-deficient pNL4-3 Luc^+^ Env [22,23]. Briefly, three million HEK293T cells were plated on T75 flasks (Corning Inc. NY, USA.). Cells were co-transfected 24 h after plating, and, after 48 h, the cell supernatant containing the virus was collected, filtered, centrifuged, and pooled together as described [23]. Purified pseudo-viruses were validated for infectivity and p24 content post-production.

### 2.8. Pseudoviral Infection Inhibition Assay for MVN Fusions

A total of 7500 HOS.T4.R5 cells were seeded in 96-well plates on day one, and 24 h later, the YU2, JRFL, and Bal.01 pseudo-viruses were added onto the plated cells in the presence or absence of MVN or its variants at various dilutions. The amount of viruses to be added to each well was determined based on capsid p24 protein and infection titer experiments, as reported earlier [23,24]. For an optimal signal, virus stocks were diluted in growth media (RPMI 1640) such that the final dilution of the virus showed a signal of 10^5^ luminescence counts for the YU2 and JRFL strains and 10^6^ luminescence counts for Bal.01. Bal.01 showed a higher infectivity profile based on being a lab-adapted strain and having a much higher infectivity profile [25,26]. After virus and compound addition, the plates were incubated for 24 h at 37 °C, followed by a media change process. After another 24 h, the luciferase activity was detected as described earlier [25].

### 2.9. Competition of 2G12 by MVN and Its Mutational Variants

Initially, 50 ng of gp120 was immobilized on high-binding polystyrene ELISA plates overnight at 4 °C, followed by blocking with 3% BSA for two hours at room temperature on a rocker. The blocked plates were rinsed three times with PBS-T (PBS and 0.05% Tween 20). ELISA experiments were performed by two different methods. In the first method, various concentrations of MVN or its variants were pre-incubated and allowed to bind to gp120 on the plate for two hours. After two hours, plates were washed with 1X PBS-T once, and 50 ng of 2G12 was added to all the wells. The bound 2G12 was detected using anti-human HRP. In the second method, 50 ng of 2G12 (dilution factor 1:3000) was added onto the plate simultaneously with increasing concentrations of MVN or its variants (1000 nM–0.4 nM) and incubated for two hours, followed by three PBS-T washes. Anti-human HRP was added onto the plate and incubated for another hour. The plate was washed three times with PBS-T and one more time with PBS before the addition of OPD. 2G12, loaded alone (without MVN protein), was used as a positive control for the experiment, and PBS was used as a negative control.

## 3. Results

### 3.1. Mutational Analysis of MVN Variants by Calorimetry

**Figure 2:**
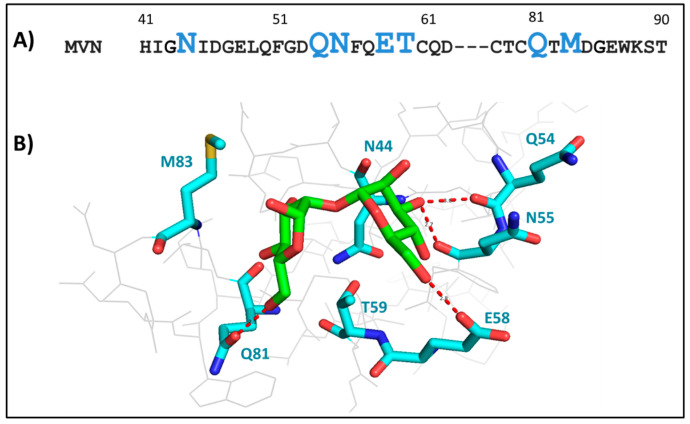
Amino acid sequence of mannobiose-binding site of MVN and structural analysis of mannobiose-bound structure of MVN (PDB ID: 2YHH). (**A**) Polar, non-polar, and charged amino acids were singly substituted by alanine. Polar (N44, Q54, N55, T59, Q81), non-polar (M83), and charged (E58) are shown in blue. (**B**) Stick representation shows mannobiose on top of various residues such as N44, T59, Q81, M83, E58, Q54, and N55. No hydrogen bonding interaction is seen with residues N44 or T59. Direct hydrogen bonding interaction (bond distance of 2.5 Å) is seen with the side chain of Q81, but not with M83. Also, direct hydrogen bonding interaction (bond distance of 2.8 Å) is seen with the side chain of E58 and the main chain carbonyl groups of Q54 (bond distance of 3.0 Å). Carbonyl oxygen of N55 lies at a bond distance of 2.7 Å and 2.9 Å from mannobiose oxygen, but at an angle of 120°. Structures were prepared using Pymol (Schrodinger Inc.).

Sequence and structural assessment of Microvirin showed that a majority (five) of the residues in the mannobiose-binding site of MVN were polar, including two glutamines (Q54, Q81), one threonine (T59), and two asparagines (N44, N55). Additionally, there was one non-polar residue (M83) and only one charged residue (E58) (Figure 2A). N-terminal residues (Q54, N55, E58) showed four polar contacts with mannobiose (Figure 2B). Central polar residues T59 and N44 showed no polar contact, whereas C-terminal residue (Q81 and M83) showed one polar contact with mannobiose (Figure 2B).

**Figure 3:**
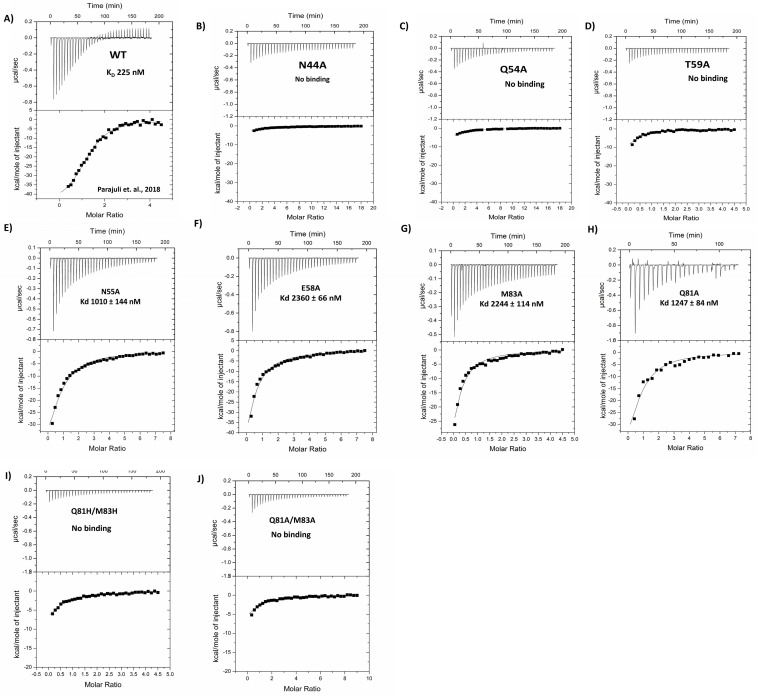
Isothermal Titration Calorimetry (ITC) analysis of the binding of MVN and its variants to gp120. Exothermic thermograms show the thermodynamics of binding. Gp120 protein (1.5 mL 3–5 µM)) in the sample cell was titrated with WT MVN (30 µM), N44A (400 µM), Q54A (200 µM), T59A (400 µM), Q81H/M83H (300 µM), Q81A/M83A (400 µM), Q81A (80 µM), M83A (140 µM), N55A (60 µM), and E58A (140 µM) at 25 °C. All experiments were performed on the (VP-ITC) system (MicroCal TM, GE Healthcare, Freiburg), and data were integrated using a one-site model.

Since mannobiose’s interaction with Microvirin provides a tentative footprint of how Microvirin interacts with glycosylation sites in glycoprotein gp120, based on this assumption, seven single alanine mutants of Microvirin were recombinantly produced, namely N44A, Q54A, T59A, N55A, E58A, M83A, Q81A, and evaluated for gp120 interaction. Three mutants (N44A, Q54A, and T59A) showed no detectable binding to gp120 compared to wild-type Microvirin (Figure 3 and Table 1). Four mutations (N55A, E58A, M83A, Q81A) showed slight reductions in the binding affinity with gp120 (Figure 3 and Table 1), with approximately 5–10-fold loss of binding, indicating that these amino acids, although not essential, are important for interaction. Our previous investigation showed that the double mutation Q81K/M83R greatly stabilized the MVN:gp120 complex by lowering the *K_D_* by almost 6-fold [14]. Since lysine (K) and arginine (R) are both positively charged amino acids, we hypothesized that Q81K/M83R would be able to introduce new hydrogen bonds with mannobiose. These residues have side chains with higher degrees of freedom that can form more alternate conformations to stabilize these hydrogen bonds with mannobiose [14]. To test the hypothesis, histidine, a positively charged residue with a rigid side chain and a limited degree of freedom, was introduced at both positions. We evaluated whether altering the flexibility without affecting the charge would influence mannobiose binding. Therefore, a double mutant Q81H/M83H was introduced and tested. A double mutant Q81A/M83A was also created as a control and tested. ITC binding analysis showed much weaker binding of purified double mutant Q81H/M83H (Figure 3). The observed data confirm that a positive charge accompanied by a higher degree of freedom is more desirable for a much more stabilized interaction at the 81st and 83rd positions as well.

### 3.2. Epitope (Hotspot Residue) Confirmation by SPR Binding Analysis

**Figure 4:**
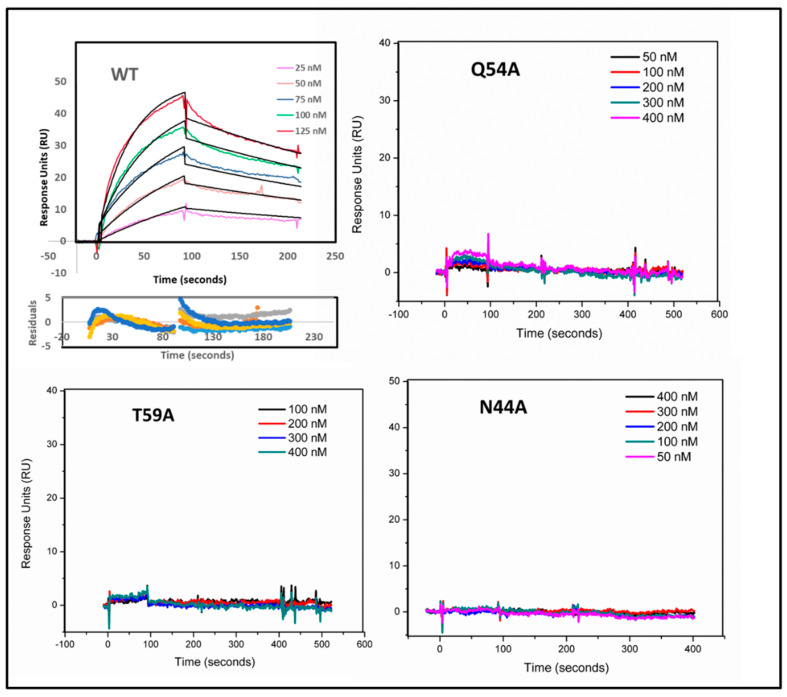
Sensorgram signals for various MVN mutants flowed over WT gp120 protein immobilized on a CM5 chip. Various concentrations of MVN mutants were passed over gp120 immobilized on the CM5 surface. Immobilized Vectibix was used as a negative control surface. Analyte proteins were associated for 90 s, followed by injection of running buffer for 120+ s. Signals show the net binding response after double subtraction of signal (buffer control + negative control surface).

To validate the hotspot residues identified via calorimetry, binding interaction analysis was performed using the surface plasmon resonance method (Biacore3000). Post-optimization, the binding of MVN and its variants was measured when they were flowed over a CM5 chip surface on which gp120 was immobilized. The analyte (MVN variant) flow rate was 50 µL/min [14,15]. For almost 500 RU of immobilized gp120, a response of almost 50 RU was observed for WT MVN. Accounting for molecular weight (120 kDa for gp120 and ~15 kDa for MVN), a binding of almost 1:1 was observed based on the response. The K_D_ value for MVN binding to the gp120 was determined to be 126 nM [15]. As expected, mutants N44A, Q54A, and T59A showed no detectable binding (Figure 4), confirming the calorimetric results.

### 3.3. Infection Inhibition Analysis of MVN Mutants

Purified MVN variants were tested for their potency in biological assays on three different strains of HIV-1 viruses, JRFL, YU2 and Bal.01, which expressed glycosylated gp120 on their surfaces. Sequence alignment was performed to compare the binding sites specific to MVN in the three strains of gp120 (Figure S3). These are the glycosylation sites (N262, N332, N448) that were recently identified [15]. All three strains of viruses had these glycosylation sites. HOS.T4.R5 cells were exposed to HIV-1 pseudoviruses (JRFL, YU2 and Bal.01) with serial dilutions of the MVN proteins and its variants. A dose-dependent inhibition of infection was seen for all strains of HIV-1 by MVN and its variants (N55A, E58A, Q81A, M83A,) but not for the variants N44A, Q54A, and T59A (Table 1 and Figure S4). The Bal.01 pseudotyped virus was found to be more sensitive to Microvirin compared to the JRFL or YU2 strains. The level of sensitivity to corresponding MVN mutations varied significantly depending on the strain type. Bal.01 exhibited a ~2–3.5-fold change in sensitivity for variants N55A, E58A, Q81A, and M83A, whereas YU2 and JRFL exhibited up to >20-fold changes in viral sensitivity for the four functional mutants. These results also correlate to the ITC data that show approximately 5- to 10-fold losses of function for the variants N55A, E58A, Q81A, and M83A compared to the wild type. The correlation of biological data with binding data argues that MVN binds to an HIV-1 virus in a similar fashion as it would bind to a recombinant protein.

### 3.4. Competition of 2G12 by MVN Variants

Earlier evidence suggested that MVN and 2G12 epitopes in gp120 overlap [15]. Since multiple variants of MVN were made, the MVN variants were tested for competition by 2G12 to ensure that the mutations introduced would not alter the 2G12 competition profiles of MVN variants. The results (Figure S5) showed that the variants N55A, E58A, Q81A, M83A, and WT, but not N44A, Q54A, and T59A, competed with 2G12 binding. No significant difference was seen regarding whether MVN was simultaneously competed with 2G12 (Figure S5A) or added separately (MVN preincubated with gp120 followed by 2G12 addition) (Figure S5B). In summary, our work helps us identify the hotspots of MVN that are involved in virus engagement and neutralization.

## 4. Discussion

Lectins such as Cyanovirin-N utilize a multivalent mode of interaction to engage glycans [8]. The high affinity shown by these glycans is due to their ability to engage multiple glycan sites via their multiple domains. In contrast, Microvirin, which is structurally very similar to Cyanovirin, is monovalent. Structural analysis (Figure 1D) shows that the second potential engagement site of Microvirin is primarily incapacitated by the presence of the amino acid residues proline (P2) and aspartate (D102), which together disrupt the corresponding mannobiose-binding pocket. The distinctive cyclic structure of main-chain proline causes conformational rigidity and less flexibility, preventing cavity formation for mannobiose binding in Microvirin (Figure 1D). In CVN, this behavior is not seen due to the presence of lysine (K3) at the corresponding position, which provides much higher flexibility (Figure 1B).

Mutational, computational, and structural studies performed with Cyanovirin-N have shown the importance of charged residues for its engagement with glycans [16,17,18]. For instance, charged residues E41 and R76 (Figure 1A) in CVN seem critical for high-affinity binding interaction with mannobiose [16,17]. Simulation studies show that R76 has no conformational preference for mannobiose, which further supports its critical role in locking ligands in a bound state [17]. Even at the low-affinity binding site of Cyanovirin-N, charged residues such as lysine (K3) and glutamate (E23) are critical for mannobiose binding (Figure 1B) [18].

On the other hand, the only charged residue E58 (Figure 1C) present at the corresponding region of Microvirin (similar to R76 at the high affinity binding site of CVN Figure 1A), although being important, does not seem essential. This is evident since the E58A mutation does not fully abolish binding (Table 1 and Figure 3). Surprisingly, not every residue that showed a drastic effect for an alanine substitution on calorimetric analysis had a direct hydrogen bonding interaction with mannobiose. For instance, no direct hydrogen bonding was seen for essential residues N44 and T59 with mannobiose (Figure 2B). N44 sits in the pocket facing inward at a distance of 6.2 Å from the center of mannobiose, apparently functioning as a space filler providing stability to the pocket. T59 projects away from the pocket center at nearly 90 degrees from the side chain of N44 at a distance of 4.8 Å from the center of mannobiose. The combined presence of N44 and T59 provides stability to the pocket for mannobiose, which explains why a mutation for either one of them to an alanine completely disrupts binding, possibly due to pocket destabilization.

A direct hydrogen bonding interaction (2.8 Å) was seen with E58 (Figure 2B); however, scanning it with alanine did not impact binding significantly, although a modest reduction in affinity was seen (Table 1). A hydrogen bonding interaction was also seen between mannobiose and Q54 (bond distance of 3.0 Å, shown in red dotted lines) (Figure 2B). However, this interaction was not with the side chain, but the main chain carbonyl group (Figure 2B). Also, the side chain of Q54 is not involved in any interactions with the mannobiose and lies at a distance of >6 Å. Given that the bonds are with the main chain, mutational variations that only alter the side chain should not impact the binding with gp120. Yet, the alanine mutation (Q54A) was detrimental to this interaction, which is surprising. A possible rationale might be that alanine mutation at this position may change the bond angle (not optimal for hydrogen bonding), which in turn may destabilize the mannobiose at this site. It is also possible that Q54 indirectly assists other residues in mannobiose interaction.

The main chain carbonyl group of N55 is also at a proximal distance from two hydroxyl groups of mannobiose (2.7 Å and 2.9 Å, shown in blue dotted lines), which is close enough to form two hydrogen bonds with main-chain and side-chain groups. However, their electron densities do not overlap each other directly, but share a bond angle of 60 and 90 degrees (Figure 2B), which is not optimal for hydrogen bonds. At this point, although we can negate the possibility of hydrogen bonds with N55 due to bond angles, we can certainly expect to see van der Waals interaction at such a close distance. This is evident from our calorimetric analysis, where a 4-fold weaker binding was seen with the N55A mutant compared to the wild-type protein (Figure 3 and Table 1).

In addition, the strong correlation of biophysical data with biological data greatly emphasizes the similarity of gp120 structural dynamics between a monomeric and virological context when present as a heterotrimer. Microvirin mutations that are insensitive or have lower responses to gp120 binding showed poor efficacy in all three virus subtypes (Bal.01, YU2, and JRFL). While the level of sensitivity was different depending on virus type, the correlation of the trend with respect to Microvirin mutation showed similarities in the interaction. The varying response can be attributed to sequential differences in regions other than gp120, as well as post-translational modifications that are different in various virus types. Finally, the monoclonal antibody (2G12) that significantly overlapped the Microvirin binding site [15] was fully competed out by Microvirin functional variants with increasing concentrations, whereas the inactive MVN variants (Q54A, N54A and T59A) were not able to fully compete out 2G12. These results also highlight the essential roles of three residues (Q54A, N54A and T59A) in gp120 interaction.

## 5. Conclusions

In summary, this study highlights the roles of both uncharged polar and non-polar residues in the monovalent glycan engagement of Microvirin with glycoprotein gp120. While residues like N55, Q54, and E58 show direct hydrogen bonding based on crystal structure, the pocket stabilizing residues N44 and T59 seem to be critical for retaining glycan-binding functionality.

## Figures and Tables

**Table 1:** Inhibition of infection of lab adapted strain of HIV-1: Inhibition of HOS.T4.R5 cell infection by YU2, JRFL, and Bal.01 strains in the presence of MVN and its variants (N55A, E58A, Q81A, M83A, N44A, Q54A, and T59A) shown in columns 3, 4, and 5. 2G12 competition with MVN and its variants shown in columns 6 and 7. Gp120 was coated on an ELISA plate followed by addition of MVN and its variants. (Column 5) 2G12 added simultaneously with increasing concentrations of MVN or its variants, or (Column 6) MVN or its variants pre-incubated with gp120 on the plate for two hours followed by addition of 50 ng of 2G12.

Mutants	KD Calorimetry ITC (µM)	YU2 (EC_50′_ µM)	JRFL (EC_50_ µM)	Bal.01 (EC_50_ µM)	2G12 Competition (Simultaneous Addition, µM)	2G12 Competition (Preincubation, µM)
WT	0.225	0.10 ± 0.01	0.1 ± 0.02	0.14	0.085	0.05
N44A	ND	ND	ND	ND	ND	ND
Q54A	ND	ND	ND	ND	ND	ND
T59A	ND	ND	ND	ND	ND	ND
N55A	1.01	0.54 ± 0.07	0.5 ± 0.04	0.32	0.27	0.18
E58A	2.36	0.56 ± 0.1	0.30 ± 0.04	0.43	0.17	0.15
M83A	2.24	1.8 ± 0.6	1.9 ± 0.25	0.46	0.17	0.2
Q81A	1.25	2.8 ± 0.2	1.2 ± 0.3	0.21	0.19	0.12
Q81A/M83A	ND	NP	NP	NP	NP	NP
Q81H/M83H	ND	NP	NP	NP	NP	NP

ND: not detected; NP: not performed.

## Data Availability

The structures currently available in databases were used for structural comparison of mannose binding sites. A list of the PDB structures used are as follows: Cyanovirin-N (1IIY), Microvirin (2YHH), Actinohivin (4DEN), Concanavalin-A (5WEY), Griffithsin (2HYQ), 2G12 antibody (6MNF), Narcissus lectin (3DZW).

## Supplementary Materials

The following supporting information can be downloaded at: https://www.mdpi.com/article/10.3390/v17010082/s1. Figure S1: Sequence and structural comparison of CVN and MVN and their lectin-binding sites. Figure S2: Figure comparing the mannose-binding sites of various lectins and monoclonal antibodies. Figure S3: Analysis of MVN and its mutants across various virus strains. Figure S4: Infection of inhibition of various HIV-1 strain with MVN WT and mutants. Figure S5: 2G12 competition by MVN and its variants. Table S1: Interaction details of mannobiose with various lectins and mAbs. References [27,28,29] are cited in the Supplementary Materials.

## Abbreviations

Env: envelope glycoprotein; MPER: membrane proximal external region, a 20-amino-acid peptide sequence (DKWASLWNWFEITEWLWYIK), also a subdomain of gp41; Trp3: truncated version of MPER with sequence DKWASLWNW; CVN: Cyanovirin, a bivalent lectin; MVN: Microvirin, a monovalent lectin; MVN-DLI: recombinant fusion of MVN with Trp3 connected by linker, with a double mutation introduced to improve Microvirin potency; DLI: dual-acting lytic inhibitor; NL4.3: A strain of HIV-1 subtype C highly prevalent in the South African population (this strain is more optimized for performing escape studies in lab settings to create resistance mutations); YU2: HIV-1 subtype B isolate (YU2 is more lab adapted for virological and biophysical analysis); JRFL: HIV-1 subtype; gp120: glycoprotein 120 (outer region of Envelope protein); gp41: transmembrane glycoprotein 41 (inner domain of envelope protein); gp160: precursor of gp120 and gp41; SPR: surface plasmon resonance; ITC: Isothermal Titration Calorimetry; HEK: human embryonic kidney cells; FP: fusion peptide, a subdomain of gp41; PGT: antibody class against gp120; VRC: antibody class against gp120; OPD: *o*-phenylenediamine dihydrochloride.
