# Supplementary material for: Monovalent Lectin Microvirin Utilizes Hydropathic Recognition of HIV-1 Env for Inhibition of Virus Cell Infection
Source: Viruses. 2025 Jan 9;17(1):82. doi: 10.3390/v17010082 (PMC11768445; doi:10.3390/v17010082)
Supplement: Supplementary file 1 [file viruses-17-00082-s001.zip › viruses-3372344-supplementary.pdf]

Figure S1. Sequence and structural comparison of CVN and MVN and their lectin binding sites. (A) Sequence alignment of CVN and MVN shows 31.5 % identity and ~52% sequence similarity. Sequence alignment was performed using EMBOSS Needle Sequence Alignment tool. **(B)** Cartoon structural comparing structure of MVN (PDB ID: 2YHH; shown in green cartoon) with CVN (PDB ID: 1IIY; shown in magenta cartoon). Structurally, both lectins have similar folds except for minor variations in beta loops. **(C)** Mannobiose (*cyan sticks)* binding site of Microvirin compared with Cyanovirin-N. Residues for MVN (*green sticks*) and CVN (*pink sticks*) show a high degree of similarity in terms of positioning except for key residues (Q54 and M83 for MVN and E41 and R76 for CVN). E41 and R76 keep the CVN pocket more compact and closed unlike MVN which is more open. Structures are drawn using Pymol (Schrodinger Inc.).

A)

```
Length: 108
Identity:      34/108 (31.5%)
Similarity:    56/108 (51.9%)
Gaps:           7/108 ( 6.5%)
Score: 131.0
```

```
  1 MPNFSHTCSSINYDPDSTILSAECQARDGEWLPTELRLSDHIGNIDGELQ[MVN] 50
    :..||.||  .|.....::|::.|:..:|.:..:.:.|:..|.|:||.|:
  1 LGKFSQTC--YNSAIQGSVLTSTCERTNGGYNTSSIDLNSVIENVDGSLK[CVN] 48

 51 FGDQNFQETCQDCRLEFGDGEQSVWLVCTCQTMDGEWKSTQILLDSQIDN[MVN]100
    :...||.|||::.:|. |..|    |...|:|...::.||:|.||..|.|
 49 WQPSNFIETCRNTQLA-GSSE----LAAECKTRAQQFVSTKINLDDHIAN[CVN] 93

101 NDSQLEIG[MVN]108
    .|..|:..
 94 IDGTLKYE[CVN]101
```

B)

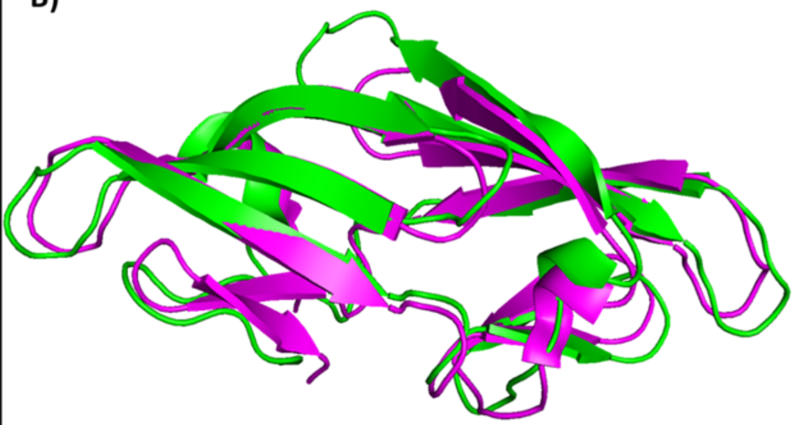

C)

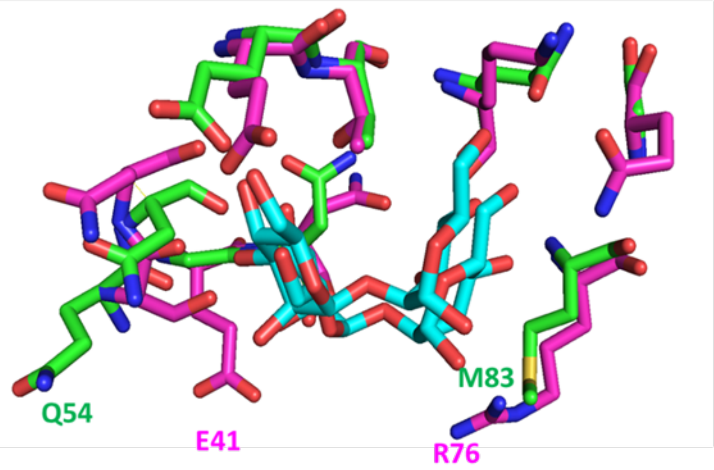

Figure S2. Figure comparing the mannose binding sites of various lectins and monoclonal antibodies. Lectin surfaces are shown as surface representation and bound mannose structures are shown in green stick model. **(A)** Mannose binding site of Concanavalin-A (Con-A) with mannobiose (PDB ID: 5WEY). Con-A only contains two polar (*violet*-Y100, N14), three charged (*yellow*-D208, R228 and D16) and a non-polar amino acids (*cyan*- L99). **(B)** Mannose binding site of Griffithsin with mannobiose (PDB ID: 2HYQ). Griffithsin contains one polar (*violet*-Y68), two charged (*yellow*-D67, D70) and two non-polar amino acids (*cyan*- G66, G90). **(C)** Structure of mannobiose bound to narcissus lectin complex (PDB ID: 3DZW). Mannobiose sits parallel to the lectin surface and forms polar contacts with three polar residues (N30, Y34 and Q26) and a charged residue (D28). **(D)** Mannose binding site of 2G12 antibody (PDB ID: 6MNF) with mannose-8[1]. Mannose-8 binds perpendicular to the surface of 2G12 and inserts deep inside the cavity. 2G12 contains four charged (*yellow*- H32, K95, D100(B), D100(D)) and four polar residues (*violet*- S53, T52, S100(B), N100(B)) all located on the mannose binding cavity. S100, D100(B) T33 and K95 form the lining of the mannose-8 binding cavity (*visible from outside)* while the rest of the residue lie deep in the pocket *(not shown)*. **(E)** Mannose binding site of Actinohivin (AVN) with mannobiose (PDB ID: 4DEN). Mannobiose sits perpendicular to the surface of actinohivin to form polar contacts with polar residues (Y116, S100, Y107, Q117 and N112) and a charged residue (D99). Structures are drawn using Pymol (Schrodinger Inc.).

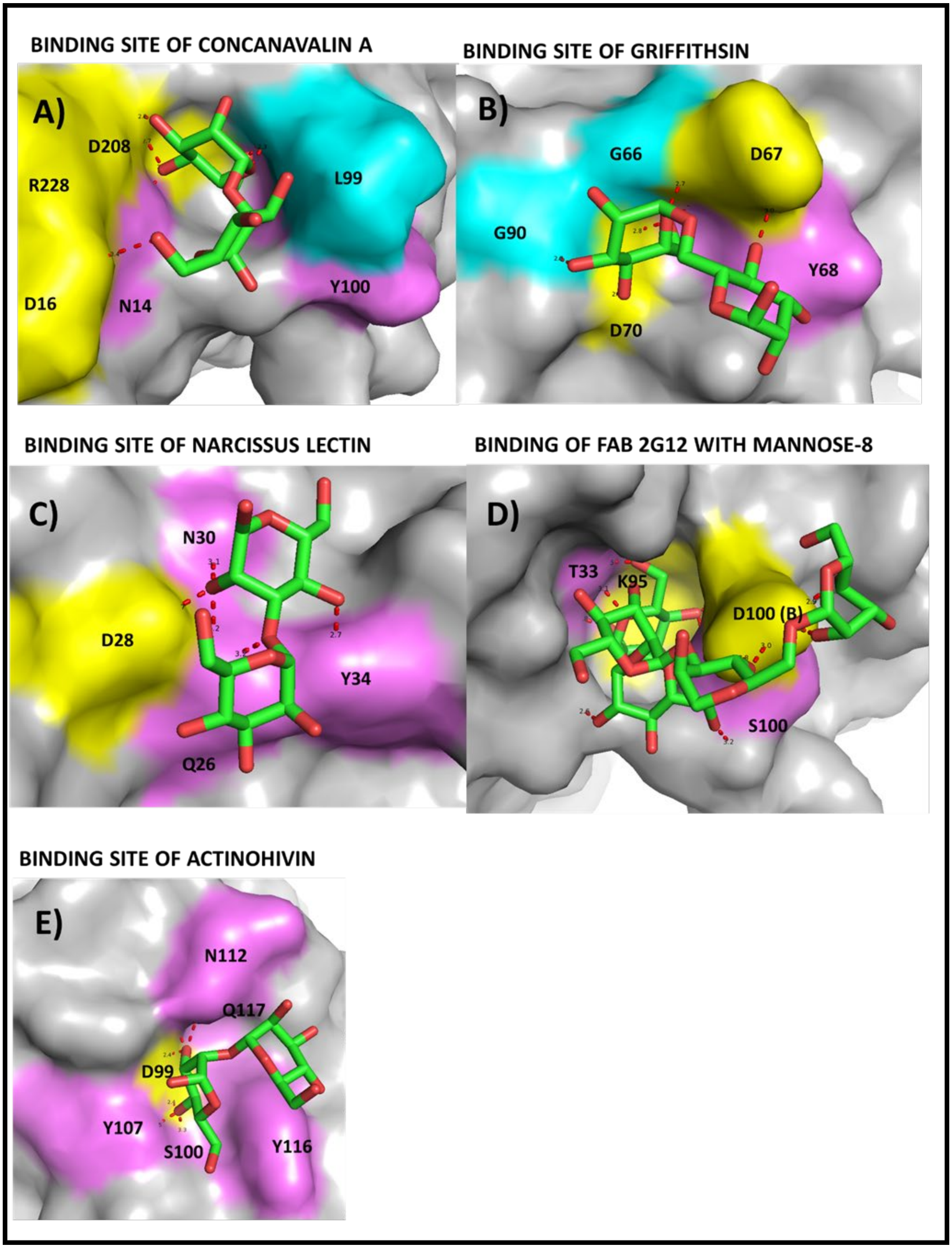
BINDING SITE OF CONCANAVALIN A
A)
D208
R228
L99
D16
N14
Y100
BINDING SITE OF GRIFFITHSIN
B)
G66
D67
G90
Y68
D70
BINDING SITE OF NARCISSUS LECTIN
C)
N30
D28
Y34
Q26
BINDING OF FAB 2G12 WITH MANNOSE-8
D)
T33
K95
D100 (B)
S100
BINDING SITE OF ACTINOHIVIN
E)
N112
Q117
D99
Y107
S100
Y116

Figure S3. Analysis of MVN and its mutants across various virus strains. Sequence alignment comparing the binding site of MVN in gp120 in three strains JRFL, YU2 and Bal.01. Recently identified glycosylation sites specific to MVN (N262, N332 and N448) are highlighted in red. All three strains of gp120 viruses have these three sites conserved.

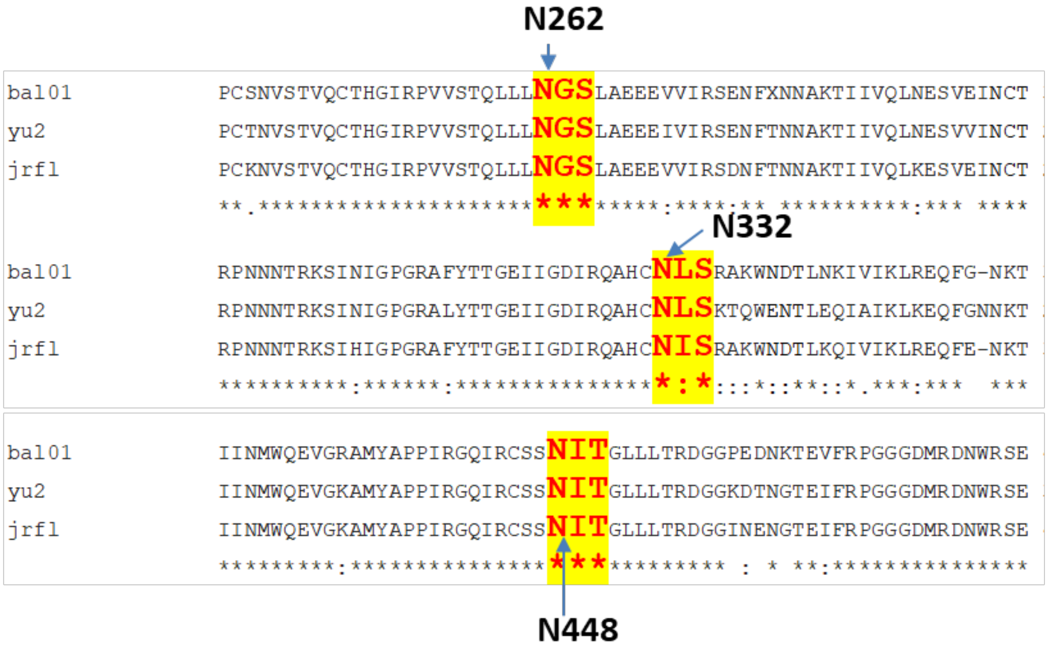

Figure S4. Inhibition of infection of lab adapted strains of HIV-1, including(**A**) (Bal.01) **(B)** YU2 and **(C)** JRFL, by MVN and its variants (N55A, E58A, Q81A, M83A). HOS.T4.R5 cells were exposed to HIV-1 Bal.01, YU2 and JRFL pseudoviruses with serial dilutions of the MVN proteins and its variants. No inhibition of infection was seen for variants N44A, Q54A and T59A. IC_{50} values were calculated using Origin v.8.1.

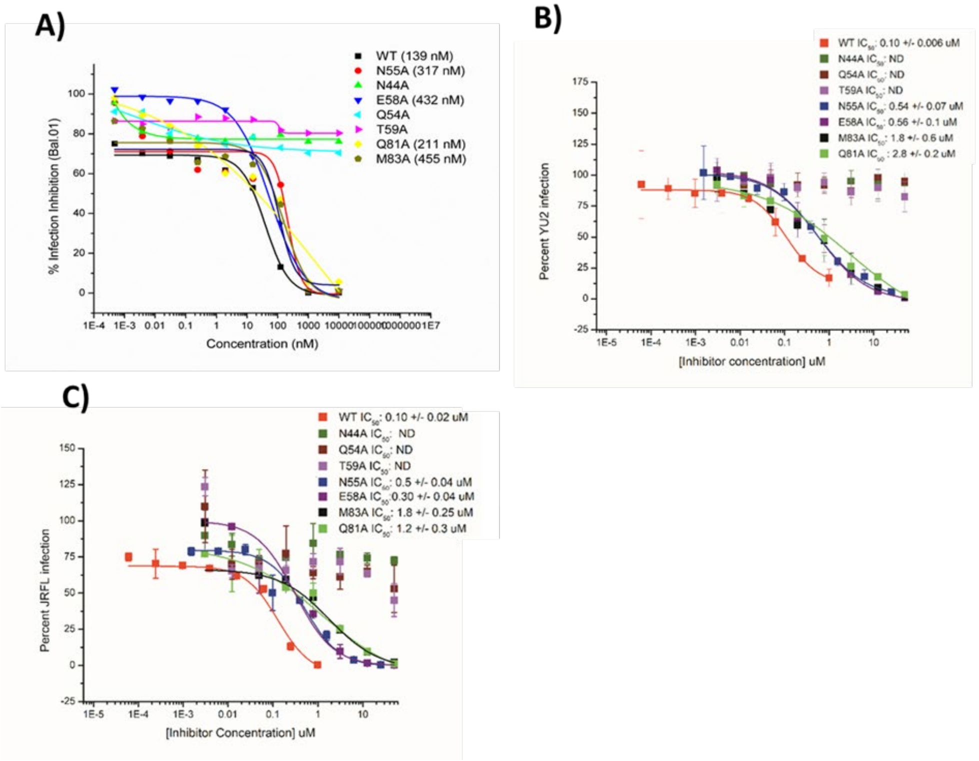

Figure S5. 2G12 competition by MVN and its variants. Gp120 (50 ng) immobilized on ELISA plates were incubated with 2G12 in the presence of MVN and its variants. **(A)** 50 ng of 2G12 (dilution factor 1:3000) was added onto the plate simultaneously with increasing concentrations of MVN or its variants (2500 nM- 0.4 nM) and incubated for two hours. **(B)** Various concentrations of MVN or its variants were preincubated and allowed to bind to gp120 on the plate for two hours followed by addition of 50 ng of 2G12. For both experiments, the bound 2G12 was detected using anti-human HRP. 2G12 loaded alone (without MVN protein) was used as positive control for the experiment and PBS was used as a negative control.

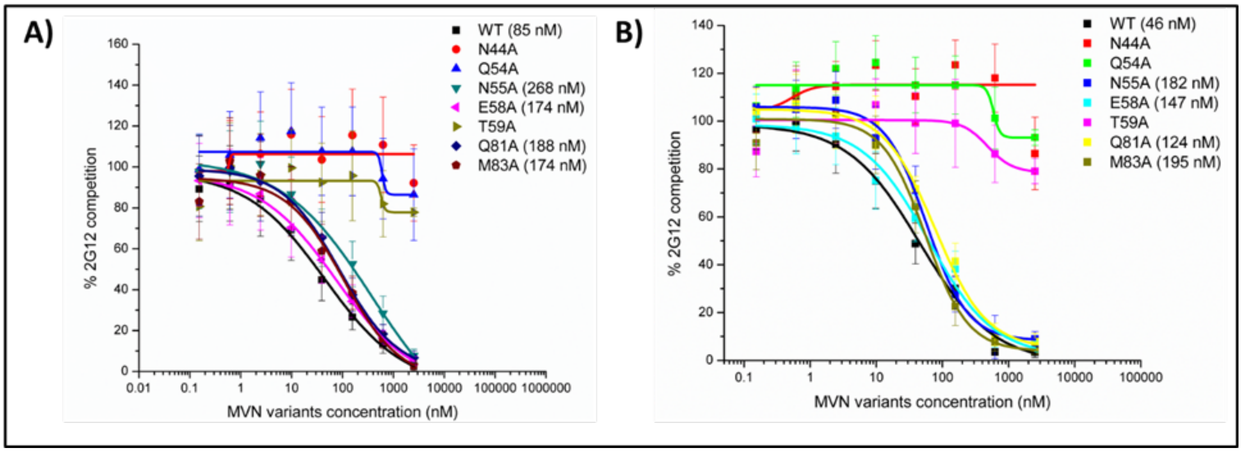

Table S1. Interaction details of mannobiose to various lectins and mAbs. Charged residues are shown in bold font and underlined.

PDB ID	Lectin	Ligand type	Charged & Polar residues	Non Polar residues	Alignment with the lectin surface	No of polar mannose involved	No of polar contact observed	Binding type
1IIY	Cyanovirin[2]	Man-Alpha 1,2, Man-Alpha	**<u>E56</u>**, T57, **<u>K74</u>**, Q78, **<u>R76</u>**, N42, S52, **<u>E41</u>**, N53	none	parallel	2	4	Multivalent
1IIY	Cyanovirin[2]	Man-Alpha 1,2, Man-Alpha	**<u>K3</u>**, Q6, T7, **<u>E23</u>**, **<u>R24</u>**, T25, **<u>D95</u>**, N93	none	parallel	1	4	Multivalent
4DEN	Actinohivin[3]	Alpha (1,2) mannobiose	N112, Q117, **<u>D99</u>**, Y107, S100, Y116	none	perpendicular	1	6	Multivalent
2YHH	Microvirin[4]	Man-Alpha 1,2, Man-Alpha	Q81, T59, N44, **<u>E58</u>**, N55, Q54	M83	parallel	2	5	Monovalent
5WEY	Concanavalin-A[5]	alpha (1,2) D-mannobiose	**<u>R228</u>**, **<u>D16</u>**, **<u>D208</u>**, N14, Y100	L99	parallel	2	8	Monovalent
2HYQ	Griffithsin[6]	6-alpha mannobiose	**<u>D70</u>**, **<u>D67</u>**, Y68	G90, G66	perpendicular	2	8	Multivalent
6MNF	2G12[1]	mannose 8	H32, **<u>K95</u>**, **<u>D100(B)</u>**, **<u>D100(D)</u>**, S53, T52, S100(B), N100(B)	none	perpendicular	4	15	Monovalent
3DZW	Narcissus lectin	mannobiose	**<u>D28</u>**, N30, Q26, Y34	none	parallel	2	6	Multivalent

REFERENCES

[1] Calarese, D. A., Lee, H. K., Huang, C. Y., Best, M. D., Astronomo, R. D., Stanfield, R. L., Katinger, H., Burton, D. R., Wong, C. H., and Wilson, I. A. (2005) Dissection of the carbohydrate specificity of the broadly neutralizing anti-HIV-1 antibody 2G12, *Proceedings of the National Academy of Sciences of the United States of America 102*, 13372-13377.

[2] Bewley, C. A. (2001) Solution structure of a cyanovirin-N:Man alpha 1-2Man alpha complex: structural basis for high-affinity carbohydrate-mediated binding to gp120, *Structure (London, England : 1993) 9*, 931-940.

[3] Hoque, M. M., Suzuki, K., Tsunoda, M., Jiang, J., Zhang, F., Takahashi, A., Ohbayashi, N., Zhang, X., Tanaka, H., Omura, S., and Takénaka, A. (2012) Structural insights into the specific anti-HIV property of actinohivin: structure of its complex with the α(1-2)mannobiose moiety of gp120, *Acta crystallographica. Section D, Biological crystallography 68*, 1671-1679.

[4] Shahzad-ul-Hussan, S., Gustchina, E., Ghirlando, R., Clore, G. M., and Bewley, C. A. (2011) Solution structure of the monovalent lectin microvirin in complex with Man(alpha)(1-2)Man provides a basis for anti-HIV activity with low toxicity, *The Journal of biological chemistry 286*, 20788-20796.

[5] Gerlits, O. O., Coates, L., Woods, R. J., and Kovalevsky, A. (2017) Mannobiose Binding Induces Changes in Hydrogen Bonding and Protonation States of Acidic Residues in Concanavalin A As Revealed by Neutron Crystallography, *Biochemistry 56*, 4747-4750.

[6] Ziółkowska, N. E., Shenoy, S. R., O'Keefe, B. R., McMahon, J. B., Palmer, K. E., Dwek, R. A., Wormald, M. R., and Wlodawer, A. (2007) Crystallographic, thermodynamic, and molecular modeling studies of the mode of binding of oligosaccharides to the potent antiviral protein griffithsin, *Proteins 67*, 661-670.
